# High-Mobility Group A1 Promotes Cardiac Fibrosis by Upregulating FOXO1 in Fibroblasts

**DOI:** 10.3389/fcell.2021.666422

**Published:** 2021-08-26

**Authors:** Qingwen Xie, Qi Yao, Tongtong Hu, Zhulan Cai, Jinhua Zhao, Yuan Yuan, Qing Qing Wu, Qi-zhu Tang

**Affiliations:** ^1^Department of Cardiology, Renmin Hospital of Wuhan University, Wuhan, China; ^2^Cardiovascular Research Institute, Wuhan University, Wuhan, China; ^3^Hubei Key Laboratory of Cardiology, Wuhan, China

**Keywords:** cardiac fibrosis, fibroblasts, high-mobility group A1, FOXO1, cardiac dysfunction

## Abstract

High-mobility group A1 (HMGA1) acts as a transcription factor in several cardiovascular diseases. However, the implications of HMGA1 in cardiac fibrosis remain unknown. Here, we investigated the impact of HMGA1 on cardiac fibrosis. A mouse cardiac fibrosis model was constructed *via* subcutaneous injection of isoproterenol (ISO) or angiotensin II (Ang II) infusion. Adult mouse cardiac fibroblasts (CFs) were isolated and cultured. CFs were stimulated with transforming growth factor-β1 (TGF-β1) for 24 h. As a result, HMGA1 was upregulated in fibrotic hearts, as well as TGF-β-stimulated CFs. Overexpression of HMGA1 in CFs aggravated TGF-β1-induced cell activation, proliferation, and collagen synthesis. Overexpression of HMGA1 in fibroblasts, by an adeno-associated virus 9 dilution system with a periostin promoter, accelerated cardiac fibrosis and cardiac dysfunction. Moreover, HMGA1 knockdown in CFs inhibited TGF-β1-induced cell activation, proliferation, and collagen synthesis. Mechanistically, we found that HMGA1 increased the transcription of FOXO1. The FOXO1 inhibitor AS1842856 counteracted the adverse effects of HMGA1 overexpression *in vitro*. HMGA1 silencing in mouse hearts alleviated Ang II-induced cardiac fibrosis and dysfunction. However, FOXO1 knockdown in mouse hearts abolished the deteriorating effects of HMGA1 overexpression in mice. Collectively, our data demonstrated that HMGA1 plays a critical role in the development of cardiac fibrosis by regulating FOXO1 transcription.

## Introduction

Cardiac fibrosis caused by various cardiac injuries is characterized by the production of excessive extracellular matrix (ECM) in the cardiac interstitium, leading to increased ventricular stiffness and diastolic dysfunction, and ultimately giving rise to heart failure (Gyongyosi et al., 2017). It is also caused by various cardiac pathophysiologic insults involving acute myocardial infarction, hypertension, and diabetes mellitus (Asbun and Villarreal, 2006). Neurohormonal activation is the main pathological feature of cardiac fibrosis during cardiac injury (Sekaran et al., 2017). Isoproterenol (ISO), a β-adrenergic agonist, could increase cardiac fibroblasts (CF) proliferation, and collagen synthesis through excessive stimulation of β-adrenergic receptors in the heart (Benjamin et al., 1989). Other neurohumoral factors, such as angiotensin II (Ang II) and transforming growth factor β1 (TGF-β1), are also increased during cardiac injury and act as key contributors to cardiac fibrosis (Cucoranu et al., 2005; Flevaris et al., 2017). Cardiac myofibroblasts come from several different sources, such as resident endothelial cells or circulating immune cells. However, research has shown that resident CFs account for the majority of activated fibroblasts during injury. Currently, the mechanism that regulates the process of cardiac fibrosis is not fully understood.

High-mobility group A1 (HMGA1), a non-histone chromatin-binding protein, is a nuclear architectural factor (Johnson et al., 1989). HMGA1 is a key regulator of a variety of fundamental biological processes, such as embryologic development, cell cycle progression, differentiation, apoptosis, inflammation, and DNA repair. Recent studies have suggested that HMGA1 is closely involved in the regulation of cardiac pathologies. HMGA1 participates in the inflammatory process of atherosclerosis, causing coronary heart disease (Schlueter et al., 2005). Inhibition of HMGA1/NF-κB signaling attenuates myocardial damage caused by coronary microembolization and improves cardiac function (Su et al., 2018). Moreover, we previously demonstrated that HMGA1 aggravated myocardial inflammation and apoptosis in a sepsis-induced cardiac injury mouse model by regulating both COX-2 and STAT3 signaling (Cai et al., 2020). In diabetic cardiomyopathy, HMGA1 inhibits autophagy by regulating the activity of the miR-222 promoter, thereby aggravating cardiac dysfunction (Wu et al., 2020). Altogether, HMGA1 could be a promising molecule in cardiac fibrosis. In the present study, we aimed to explore the role and mechanism of HMGA1 in cardiac fibrosis induced by ISO or Ang II in mice, as well as in CFs stimulated by TGF-β1. Periostin, an epithelial ligand and matricellular protein commonly expressed by fibroblasts, has been dissected by many studies as a promoter responsible for controlling gene expression in fibroblasts (Piras et al., 2016; Bao et al., 2020). Thus, in this study, we used an adeno-associated virus 9 delivery system with a periostin promoter to specifically target HMGA1 gene expression in CFs.

## Materials and Methods

### Materials

Isoproterenol (ISO) and TGF-β were purchased from Sigma-Aldrich (St. Louis, MO, United States). Antibodies against HMGA1, α-SMA, MMP9, and PCNA were acquired from Abcam. Antibodies against GAPDH, total FOXO1, and phosphorylated (P)-FOXO1 (9461P) were acquired from Cell Signaling Technology. Antibodies against collagen III were acquired from Santa Cruz Biotechnology. The GT Vision^TM^ + Detection System/Mo&Rb reagent for immunohistochemistry was purchased from Gene Technology (Shanghai, China). An Alexa Fluor 488-conjugated goat anti-rabbit secondary antibody for immunofluorescence was obtained from LI-COR Biosciences.

### Animals and Models

C57/BL6J male mice (8 weeks, 25.2 ± 2 g) were purchased from the Chinese Academy of Medical Sciences (Beijing). All of the animal care and experimental procedures were in compliance with the regulations of the National Institutes of Health Guide for the Care and Use of Laboratory Animals and were approved by the Institutional Animal Use and Care Committee at Wuhan University, China. Mice were divided into four groups in the overexpression experiment (*n* = 12 per group). The mice were given injections of either adeno-associated virus (AAV9)-HMGA1 or AAV9-NC to overexpress or knock down HMGA1. After 1 week, ISO was injected subcutaneously for 14 days (10 mg/kg for 3 days and 5 mg/kg for 11 days) to establish a mouse model of cardiac fibrosis (Jiang et al., 2017). The Ang II-induced cardiac mouse model was established as follows: The mice were injected subcutaneously with Ang II (1,000 ng/kg/min) *via* an osmotic minipump for 28 days (Zhai et al., 2018). They were then divided into four groups in the knockdown experiment (*n* = 12 per group). The mice were subjected to AAV9-shHMGA1 or AAV9-ScRNA to knock down HMGA1 1 week before Ang II infusion. The mice were divided into three groups in the reverse experiment. They were also subjected to AAV9-HMGA1 and AAV9-ShFOXO1, or AAV9-ScRNA to knock down FOXO1 1 week before Ang II infusion. At the end of the ISO or Ang II treatment period, the mice were anesthetized with sodium pentobarbital and sacrificed after echocardiography.

### Adeno-Associated Virus Vector

Recombinant AAV9-expressing mouse HMGA1 (AAV9-HMGA1), AAV9-shHMGA1, and AAV9-shFOXO were constructed by Vigene Bioscience Company (Jinan, China) as described in our previous study (Wu et al., 2020). A short, small 1,395-bp periostin promoter was used to induce fibroblast-specific gene delivery (Piras et al., 2016). A total of 60–80 μl of AAV9-HMGA1/AAV9-shHMGA1/AAV9-shFOXO or AAV9-NC/AAV9-shRNA (5.0–6.5 × 10^13^ VG/ml) was injected into the retro-orbital venous plexus of the mice 1 week before ISO or Ang II injection, as described in a previous study (Wu et al., 2020).

### Echocardiography

A Mylab 30CV ultrasound system (Esaote S.P.A., Genoa, Italy) and a 10-MHz linear array ultrasound transducer were used to assess the cardiac function of the mice, which were anesthetized with continuous 1.5–2% isoflurane inhalation. Cardiac parameters were assessed by M-mode and two-dimensional echocardiography. The following parameters were collected: left ventricular (LV) ejection fraction (EF), fractional shortening (FS), LV end-systolic diameter (LVESd), and LV end-diastolic diameter (LVEDd).

### Cell Culture and Treatments

Cardiac fibroblasts (CFs) were isolated from adult mice (6–8 weeks) as described in our previous study (Liu et al., 2020). After isolation, CFs were cultured in DMEM/F12 with 10% fetal bovine serum (FBS). Two- to four-interval passaging CFs were used. CFs were transfected with adenovirus (Ad)-HMGA1 (MOI = 30) for 8 h to overexpress HMGA1. In the gene silencing experiments, cells were transfected with 100 pmol anti-HMGA1 siRNA for 8 h. TGF-β (10 ng/ml, 24 h) was used to induce CF activation. To inhibit FOXO1, CFs were treated with AS1842856 (10 μM, MedChemExpress) for 2 h and then stimulated with TGF-β (10 ng/ml) for another 24 h.

### Western Blotting

Heart tissue and CF samples were lysed in radioimmunoprecipitation (RIPA) lysis buffer. Then, 30 μg of protein from each sample were separated by 10% SDS-PAGE. Protein was then transferred to PVDF membranes, followed by blocking with 5% milk for 1 h. Specific primary antibodies were incubated with PVDF membranes overnight. Then, the corresponding secondary antibodies were incubated with the membranes for 1 h. ECL Western blot detection kits (GE) were used to detect chemiluminescence with a LI-COR Odyssey image system. Protein expression levels were normalized to the matched GAPDH. Image Lab software from Bio-Rad (Hercules, CA, United States) was used for quantification.

### Quantitative Real-Time PCR

Heart tissue and CF samples were lysed in TRIzol reagent (Gibco, United States). Total RNA was extracted (Gibco, United States). SYBR green master mix (Bio-Rad, Hercules, CA, United States) reactions were used for PCR. Threshold cycles and melting curve measurements were used to quantify the PCR results. mRNA levels were normalized to the matched GAPDH. The sequences of the primers are shown in Table 1.

**TABLE 1:** Primer sequences used for RT-PCR.

**mRNA**	**Forward**	**Reverse**
Collagen I	AGGCTTCAGTGGTTTGGATG	CACCAACAGCACCATCGTTA
Collagen III	AAGGCTGCAAGATGGATGCT	GTGCTTACGTGGGACAGTCA
CTGF	AGGGCCTCTTCTGCGATTTC	CTTTGGAAGGACTCACCGCT
TGF-β	ATCCTGTCCAAACTAAGGCTCG	ACCTCTTTAGCATAGTAGTCCGC
GAPDH	ACTCCACTCACGGCAAATTC	TCTCCATGGTGGTGAAGACA

*Sequences listed are 5′–3′.*

### Histological Examination

Myocardial tissue was fixed with 10% formalin and then dehydrated and embedded in paraffin. After transverse sectioning into 5-μM sections, the hearts were stained with picrosirius red (PSR) to evaluate cardiac fibrosis. Image-Pro Plus software was used to measure cardiac fibrosis. The mean ratio of the collagen content to the total tissue area of each group was calculated as the collagen volume fraction. The expression of HMGA1 in the myocardium was immunolocalized using an anti-HMGA1 antibody (1:200) and visualized with a DAB-based colorimetric method.

### Immunofluorescence Staining

After treatment, CFs were washed with PBS and then fixed with 4% methanol and permeabilized in 0.2% Triton X-100. After washing with PBS, the cells were blocked with 10% goat serum for 1 h. The specific primary antibodies were incubated at 4°C overnight. A fluorescence-labeled secondary antibody was then used to incubate the cells at room temperature for 1 h. CF nuclei were stained with DAPI. An Olympus DX51 fluorescence microscope was used for observation (Tokyo, Japan).

### Cell Counting Kit-8

A CCK-8 assay kit was used to detect the proliferation of treated cells. A 100-μl cell suspension was placed in a 96-well plate, and the cells were treated according to the experimental design. Ten microliters of CCK-8 solution was added to each well and cultured in an incubator for 4 h. The absorbance at 450 nm was measured by an enzyme plate analyzer to determine the cell proliferation activity.

### Luciferase Reporter Assay

The amplified fragment of the FOXO1 3′-UTR was subcloned into the luciferase reporter vector (Promega, United States). The PGL3 basic vector was used as a negative control. Luciferase reporter constructs were packed with an adenoviral system and then cotransfected into CFs with a control plasmid, followed by the indicated stimulation: Ad-HMGA1 transfection or HMGA1 siRNA for 48 h. Then, the cells were harvested and lysed, and the Dual-Luciferase Reporter Assay Kit (Promega, United States) was used to detect the luciferase activity according to the instructions of the manufacturer.

### Statistical Analysis

SPSS software, version 21.0, was used to analyze the data. Two-way ANOVA was used to compare data between groups followed by Tukey’s *post hoc* test. Student’s unpaired *t*-test was used to compare data between two groups. Statistically significant findings were confirmed with a *p*-value < 0.05.

## Results

### High-Mobility Group A1 Was Upregulated During Cardiac Fibrosis

**FIGURE 1:**
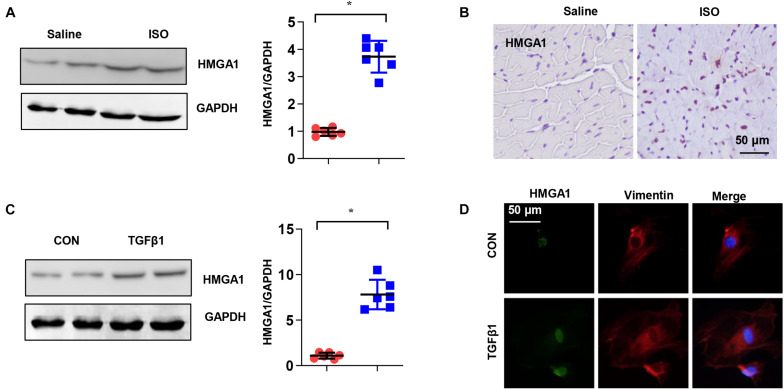
High-mobility group A1 (HMGA1) was upregulated during cardiac fibrosis. **(A)** The protein level of HMGA1 in mouse hearts after isoproterenol (ISO) insult (*n* = 6). **(B)** Immunohistochemical staining of HMGA1 in mouse hearts after ISO insult (*n* = 5). **(C)** The protein level of HMGA1 in fibroblasts after transforming growth factor-β1 (TGFβ1) insult (*n* = 6). **(D)** Immunofluorescence staining of fibroblasts after TGFβ1 insult (*n* = 5). *p < 0.05.

To explore the expression of HMGA1, we detected HMGA1 expression in ISO-induced fibrotic mouse hearts and TGF-β-stimulated CFs. HMGA1 protein and mRNA levels were increased in both fibrotic hearts and TGF-β-stimulated CFs (Figures 1A,C). Immunofluorescence staining confirmed that HMGA1, mainly located in the nuclei, was increased in fibrotic heart tissues (Figure 1B) and TGF-β-stimulated CFs (Figure 1D). These alterations indicate that an elevation in HMGA1 could be associated with cardiac fibrosis.

### High-Mobility Group A1 Overexpression Aggregated Cardiac Fibroblast Activation *in vitro*

**FIGURE 2:**
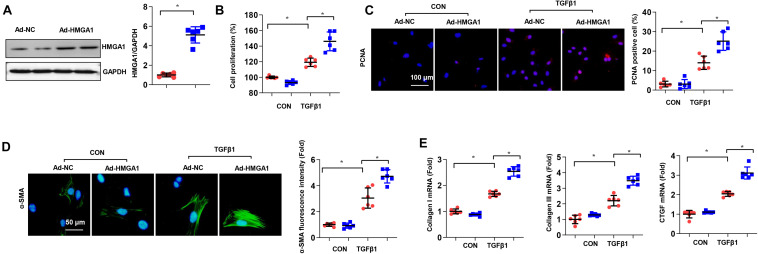
High-mobility group A1 (HMGA1) overexpression aggregated cardiac fibroblast activation *in vitro*. **(A)** The protein level of HMGA1 in fibroblasts transfected with ad-HMGA1 (*n* = 6). **(B–D)** Cardiac fibroblasts (CFs) were transfected with ad-HMGA1 and stimulated with TGFβ1 for 24 h. **(B)** Cell proliferation detected by cell counting kit 8 (CCK8) assay (*n* = 6). **(C)** Immunofluorescence staining of PCNA (*n* = 5). **(D)** Immunofluorescence staining of α-SMA (*n* = 5). **(E)** Transcription levels of collagen I, collagen III, and connective transforming tissue factor (CTGF) (*n* = 6). *p < 0.05.

After cardiac injury, stationary CFs in heart tissue are activated in myofibroblasts with active proliferation ability and collagen secretion. Therefore, we further explored the effects of HMGA1 on CFs stimulated by TGF-β *in vitro*. CFs were infected with Ad-HMGA1 to overexpress HMGA1 (Figure 2A). TGF-β stimulation increased cell proliferation, as evidenced by increased PCNA-positive cell numbers and cell proliferation (Figures 2B,C). As expected, TGF-β significantly increased the α-SMA density in CFs (Figure 2D), accompanied by increases in collagen I, collagen III, and CTGF expression at the mRNA level (Figure 2E). HMGA1 overexpression induced TGF-β-induced cell proliferation and activation (Figures 2B–D).

### High-Mobility Group A1 Overexpression Contributed to Isoproterenol-Induced Cardiac Fibrosis *in vivo*

**FIGURE 3:**
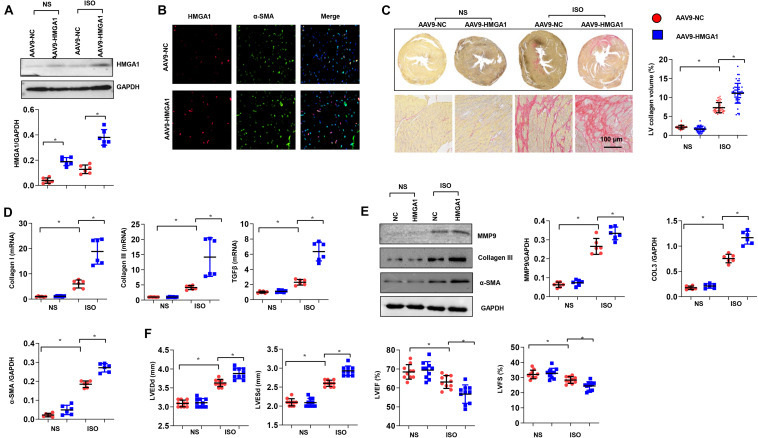
High-mobility group A1 (HMGA1) overexpression contributed to ISO-induced cardiac fibrosis *in vivo*. **(A)** The protein level of HMGA1 in mouse hearts 3 weeks after AAV9-HMGA1 injection (*n* = 6). **(B)** Immunofluorescence staining of HMGA1 and α-SMA in heart tissue post-AAV9-HMGA1 injection and ISO injection (*n* = 5). **(C)** Picrosirius red (PSR) staining image and quantitative result of left ventricular collagen volume in mouse hearts after ISO treatment (*n* = 5). **(D)** Transcription levels of collagen I, collagen III, and TGFβ1 (*n* = 6). **(E)** Protein levels of MMP9, collagen III, and α-SMA in heart tissue (*n* = 6). **(F)** Echocardiographic results in each group (*n* = 10). *p < 0.05.

To further confirm the role of HMGA1 in cardiac fibrosis, we induced HMGA1 overexpression in fibroblasts *in vivo*. HMGA1 increased at 4 weeks after AAV9-HMGA1 injection (Figure 3A). HMGA1 expression was mainly in CFs, as we used the fibroblast promoter periostin (Figure 3B). Heart tissues from mice subjected to ISO showed intense interstitial fibrosis. ISO injection induced significant cardiac fibrosis, as assessed by increased collagen deposition and increased mRNA levels of the fibrosis markers TGF-β1, collagen I, and collagen III compared with the NS group. However, HMGA1-overexpressing mice showed aggressive cardiac fibrosis with greater LV collagen deposition in the interstitial area and more upregulated mRNA levels of fibrosis markers than the NC-ISO group (Figures 3C,D). The protein expression of the fibrosis-associated molecules MMP9, collagen III, and α-SMA was also evaluated. ISO was found to induce dramatic increases in these proteins, and HMGA1 overexpression aggregated ISO-induced changes (Figure 3E). Echocardiogram results also revealed that ISO injection induced mouse cardiac dysfunction with reduced LVEF and FS and increased LVEDd and LVESd. Cardiac dysfunction was aggravated by HMGA1 overexpression (Figure 3F). Collectively, these findings suggest that HMGA1 promotes ISO-induced cardiac fibrosis and cardiac dysfunction.

### High-Mobility Group A1 Deficiency Reduced Cardiac Fibroblast Proliferation and Activation *in vitro*

**FIGURE 4:**
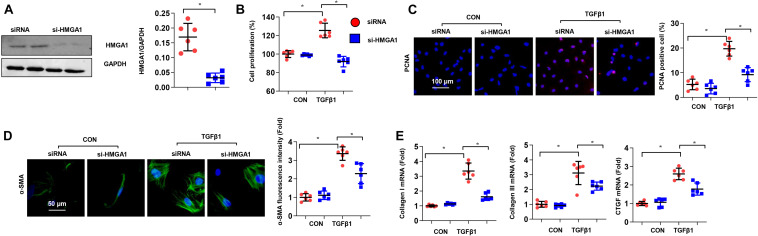
High-mobility group A1 (HMGA1) deficiency reduced cardiac fibroblast proliferation and activation *in vitro*. **(A)** The protein level of HMGA1 in fibroblasts transfected with HMGA1 siRNA (*n* = 6). **(B–D)** CFs were transfected with HMGA1 siRNA and stimulated with TGFβ1 for 24 h. **(B)** Cell proliferation detected by CCK8 assay (*n* = 6). **(C)** Immunofluorescence staining of PCNA (*n* = 5). **(D)** Immunofluorescence staining of α-SMA (*n* = 5). **(E)** Transcription levels of collagen I, collagen III, and CTGF (*n* = 6). *p < 0.05.

Next, we wondered whether HMGA1 deficiency would alleviate cardiac fibrosis pathogenesis. To address this issue, we used siRNA to knock down HMGA1 in CFs. The protein expression levels of HMGA1 decreased in CFs transfected with HMGA1 siRNA (Figure 4A). TGF-β-induced proliferation and activation of fibroblasts were also inhibited after HMGA1 knockdown (Figures 4B–D). In addition, HMGA1 silencing inhibited the transcription of collagens and CTGF (Figure 4E). Thus, targeting of HMGA1 could be a potential treatment approach for fibrosis disease.

### High-Mobility Group A1 Modulated the Expression and Phosphorylation of FOXO1

**FIGURE 5:**
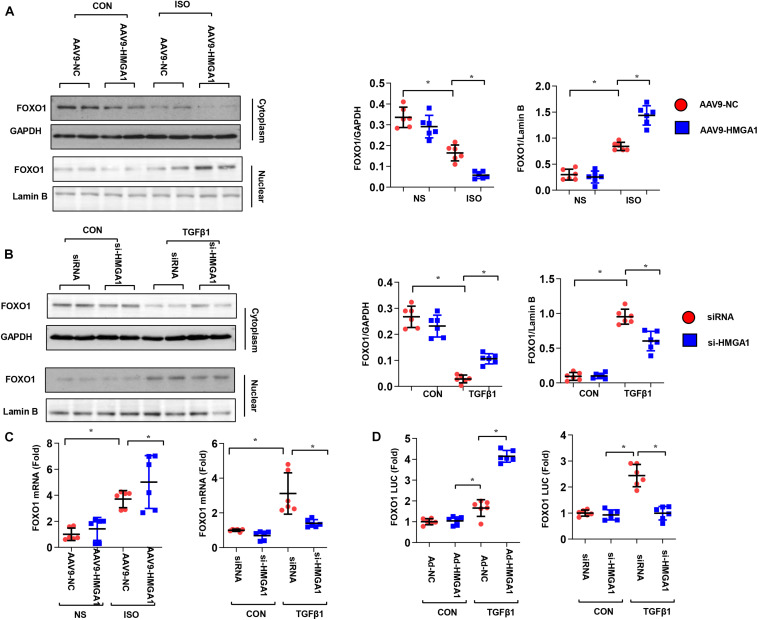
High-mobility group A1 (HMGA1) modulated the expression and phosphorylation of FOXO1. **(A)** The protein level of FOXO1 in heart tissue after AAV9-HMGA1 and ISO injection (*n* = 6). **(B)** The protein level of FOXO1 in CFs after HMGA1 siRNA and TGFβ1 treatment (*n* = 6). **(C)** The mRNA level of FOXO1 in heart tissue (left) and CFs (right) in each group (*n* = 6). **(D)** Luciferase reporter results in CFs with HMGA1 overexpression or HMGA1 silencing. *p < 0.05.

Evidence has shown that HMGA1 affects FOXO1 (Arcidiacono et al., 2018), a crucial transcription factor in the development of cardiac fibrosis (Arcidiacono et al., 2018; Xin et al., 2018). To explore the mechanisms of HMGA1 in fibrosis, we subsequently measured the total and phosphorylated levels of the FOXO1 protein. Our results showed that ISO stimulation markedly reduced FOXO1 cytoplasm levels and increased FOXO1 protein levels in nuclear mouse heart tissue (Figure 5A). HMGA1 overexpression obviously increased the nuclear level of FOXO1. Conversely, in TGF-β1-stimulated fibroblasts, and HMGA1, silencing enhanced the cytoplasmic level of FOXO1 while reducing the nuclear level of FOXO1 (Figure 5B). To test the hypothesis that HMGA1 could play a role in the transcriptional regulation of the FOXO1 gene, we detected the mRNA expression of FOXO1. As shown in Figure 5C, HMGA1 overexpression in heart tissue increased the transcription of FOXO1. HMGA1 silencing in fibroblasts downregulated FOXO1 transcription levels. We further performed reporter gene analysis in CFs. After transfecting the cells with the FOXO1-Luc reporter plasmid bearing the FOXO1 promoter sequence upstream of the luciferase reporter gene, FOXO1-Luc activity was increased in cells with HMGA1 overexpression and decreased in cells with HMGA1 silencing only under TGFβ1 stimulation (Figure 5D). This finding indicates that, during the fibrosis process, HMGA1 promotes the expression of FOXO1 by binding to the FOXO1 promoter and increases FOXO1 nuclear levels.

### FOXO1 Inhibition Abolished the Effect of High-Mobility Group A1 Overexpression *in vitro*

**FIGURE 6:**
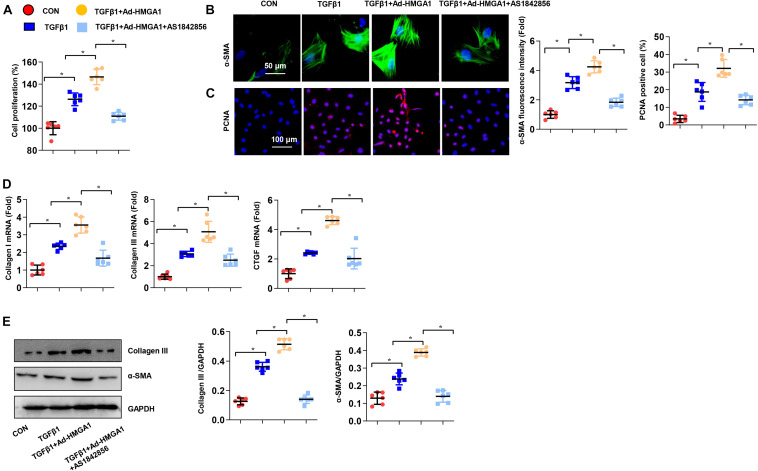
FOXO1 inhibition abolished the effect of HMGA1 overexpression *in vitro*. CFs were treated with AS1842856 (10 μM) and then treated with TGFβ1 for 24 h. **(A)** Cell proliferation detected by CCK8 assay (*n* = 6). **(B)** Immunofluorescence staining of α-SMA (*n* = 5). **(C)** Immunofluorescence staining of PCNA (*n* = 5). **(D)** Transcription levels of collagen I, collagen III, and CTGF (*n* = 6). **(E)** Protein levels of collagen III and α-SMA (*n* = 6). *p < 0.05.

To further investigate whether the effects of HMGA1 depend on FOXO1, we used AS1842856 to inhibit FOXO1 in CFs. The CCK-8 assay showed that HMGA1 overexpression promoted fibroblast proliferation, but the inhibition of FOXO1 significantly reduced the proliferative effect (Figure 6A). The inhibitory effect of FOXO1 inhibitors on fibroblast proliferation was further verified by PCNA staining (Figure 6B). In cellular immunofluorescence staining, the expression of α-SMA in fibroblasts was enhanced by TGF-β and HMGA1, while the expression of α-SMA was inhibited by the FOXO1 inhibitor (Figure 6C). Correspondingly, the expression of fibrosis-related genes and proteins decreased with the inhibition of FOXO1 (Figures 6D,E). In summary, HMGA1 affects CF activation and function *via* FOXO1.

### High-Mobility Group A1 Knockdown Alleviated Cardiac Fibrosis and Dysfunction Induced by Angiotensin II in Mice

**FIGURE 7:**
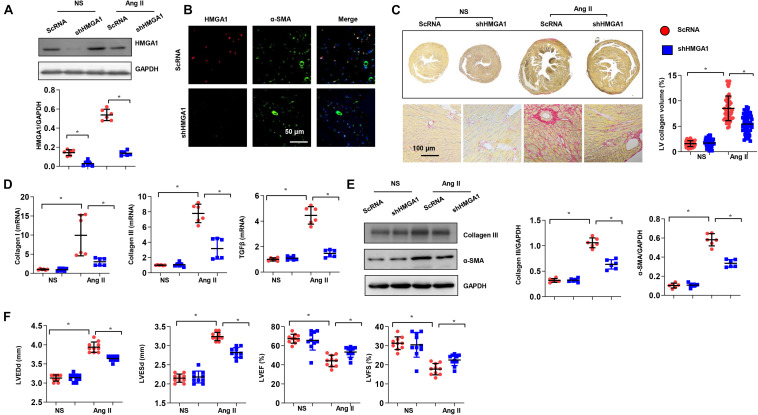
High-mobility group A1 (HMGA1) knockdown alleviated cardiac fibrosis and dysfunction induced by angiotensin II (Ang II) in mice. **(A)** The protein level of HMGA1 in mouse hearts 3 weeks after AAV9-shHMGA1 injection (*n* = 6). **(B)** Immunofluorescence staining of HMGA1 and α-SMA in heart tissue post-AAV9-shHMGA1 injection and Ang II infusion (*n* = 5). **(C)** PSR staining image and quantitative result of left ventricular collagen volume in mouse hearts 28 days after Ang II infusion (*n* = 5). **(D)** Transcription levels of collagen I, collagen III, and TGFβ1 (*n* = 6). **(E)** Protein levels of collagen III and α-SMA in heart tissue (*n* = 6). **(F)** Echocardiographic results in each group (*n* = 10). *p < 0.05.

To further confirm the role of HMGA1 in cardiac fibrosis, we silenced HMGA1 in an Ang II-induced cardiac fibrosis mouse model. AAV9-shHMGA1 injection induced a sharp reduction in HMGA1 expression after 28 days of Ang II infusion (Figure 7A). The expression of HMGA1 was coincident with the expression of α-SMA, indicating that HMGA1 was downregulated in CFs in heart tissue after Ang II infusion (Figure 7B). Chronic Ang II infusion for 28 days induced significant fibrosis in male C57BL/6 mice, as demonstrated by a significant increase in collagen volume and higher expression of fibrotic markers compared with controls (Figures 7C–E). HMGA1 knockdown dramatically inhibited Ang II-induced cardiac fibrosis, as demonstrated by significant decreases in collagen volume and expression of fibrotic markers (Figures 7C–E). Echocardiography results revealed that mice in the HMGA1 knockdown group exhibited improved cardiac function with higher LVEF and FS and reduced LVEDd and LVESd in response to Ang II infusion (Figure 7F). Considered together, these data suggest that HMGA1 knockdown leads to alleviated cardiac dysfunction in mice challenged with Ang II.

### FOXO1 Inhibition Counteracted High-Mobility Group A1-Induced Cardiac Dysfunction

**FIGURE 8:**
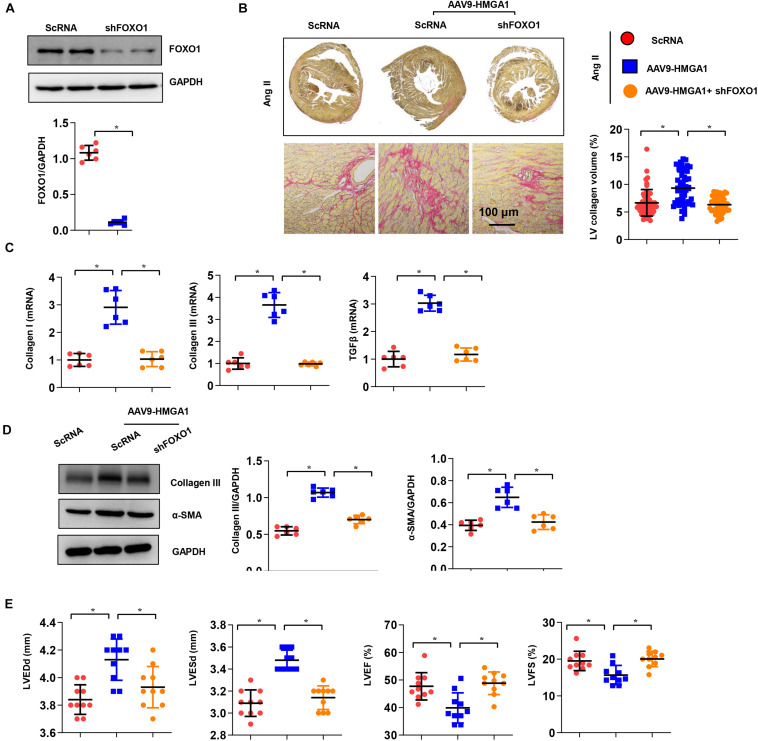
FOXO1 inhibition attenuated HMGA1-induced cardiac dysfunction. **(A)** The protein level of FOXO1 in mouse hearts 3 weeks after AAV9-shFOXO1 injection (*n* = 6). **(B)** PSR staining image and quantitative result of left ventricular collagen volume in mouse hearts 28 days after Ang II infusion (*n* = 5). **(C)** Transcription levels of collagen I, collagen III, and TGFβ1 (*n* = 6). **(D)** Protein levels of collagen III and α-SMA in heart tissue (*n* = 6). **(E)** Echocardiographic results in each group (*n* = 10). *p < 0.05.

Next, we investigated whether FOXO1 was essential for the effects of HMGA1 *in vivo*. Mice were subjected to both AAV9-shFOXO1 and AAV9-HMGA1 injections and then infused with Ang II for 28 days. AAV9-shFOXO1 induced a significant decrease in FOXO1 expression 28 days after Ang II infusion (Figure 8A), and the fibrotic effect of HMGA1 in the heart induced by Ang II was counteracted by FOXO1 knockdown, as evidenced by the decreased LV percentage fibrosis and the downregulated mRNA expression of collagen I, collagen III, and TGF-β1 (Figures 8B,C). The protein expression of collagen III and α-SMA was also reduced in mice subjected to both AAV9-shFOXO1 and AAV9-HMGA1 injections (Figure 8D). Additionally, cardiac function in mice injected with both AAV9-shFOXO1 and AAV9-HMGA1 was also improved compared with mice injected with only AAV9-HMGA1 (Figure 8E). These results further suggested that FOXO1 is the target of HMGA1 involved in cardiac fibrosis.

## Discussion

We previously provided data indicating that HMGA1 could play an important role in sepsis-induced cardiomyopathy and diabetic cardiomyopathy (Wu et al., 2020). In the present study, we showed that HMGA1 promoted ISO- or Ang II-induced cardiac fibrosis and dysfunction *in vivo*. HMGA1 knockdown blocked the activation and function of CFs. Furthermore, HMGA1 regulated fibroblast activation and function by promoting the transcription of FOXO1.

In the progression of all types of cardiac injury, such as hypertension, myocardial infarction, or diabetes, activated neurohumoral factor trigger CFs to differentiate into active cardiac myofibroblasts (Burke et al., 2019; Gorski et al., 2019; Huang et al., 2019). Myofibroblasts are widely considered to be the cause of cardiac fibrosis, and their excessive ECM secretion directly leads to the formation of scar tissue (Leask, 2015). They also express the highly contracted protein α-SMA, collagens, and connective transforming tissue factor (CTGF), which reshape the surrounding ECM. HMGA1 expression is very high during embryogenesis, while it is negligible in normal adult tissues (Reeves, 2001). In our study, we showed that the expression level of HMGA1 was upregulated in CFs after stimulation. The high expression of HMGA1 in the heart seems harmful. We previously revealed that HMGA1 increased cardiomyocyte inflammation in a sepsis-induced cardiomyopathy model (Cai et al., 2020); HMGA1 promotes cardiac dysfunction in diabetic cardiomyopathy (Wu et al., 2020). Consistently, we found that HMGA1 increased fibroblast activation and collagen synthesis, leading to aggregated fibrosis and cardiac dysfunction.

FOXO1 is involved in a broad range of cellular processes, including cell cycle arrest, DNA repair, apoptosis, oxidative stress, and glucose metabolism, as well as fibrosis. During the fibrosis process, FOXO1 is upregulated. TGF-β1 can promote FOXO1 transcription by activating p300 (Bugyei-Twum et al., 2014). Ang II also promotes the transcriptional activity of FOXO1 and increases FOXO1 activation (Li et al., 2019). During cardiac injury, overloaded neurohumoral factors increase FOXO1 activity, which acts as a pathological factor. FOXO1 directly binds to MMPs and the CTGF gene promoter and increases their transcription (Musikant et al., 2019). Moreover, FOXO1 increases fibroblast activation and proliferation *via* the p21-cell cycling pathway (Xin et al., 2018). The main way to regulate FOXO1 activity is the phosphorylation of Akt at three different sites (T24, S256, and S319), which inhibits their interaction with DNA and promotes their nuclear export and subsequent degradation by the proteasome (Eijkelenboom and Burgering, 2013). In our study, we found that HMGA1 increased the transcription of FOXO1. Moreover, HMGA1 also increased the nuclear level of FOXO1, further enhancing the transcriptional regulation of FOXO1-targeted genes. Previous studies have reported the relationship of HMGA1 and FOXO1 in many other cell types (Chiefari et al., 2018). HMGA1 increases FOXO1 mRNA and protein expression in cultured HepG2 cells and hepatocytes by combining with the FOXO1 gene promoter (Arcidiacono et al., 2018). HMGA1 is essential for FOXO1-induced IGFBP1 gene expression and, thus, participates in the insulin-mediated pathway (Chiefari et al., 2018). HMGA1 seems to affect cardiac fibrosis by relying on FOXO1. When we inhibited FOXO1 in both *in vitro* and *in vivo* experiments, the deteriorating effects of HMGA1 were counteracted.

It is worth noting that HMGA1 did not affect the promoter activity of FOXO1 only under physical status (Figure 5D). CFs secrete endogenous TGF-beta 1, which induces basal fibroblast activation. In our study, HMGA1 overexpression or knockdown also did not affect the CF phenotype. We previously reported that HMGA1 could regulate the activity of different transcription factors under different conditions (Cai et al., 2020). Thus, further study about how HMGA1 detaches the promoter region of FOXO1 under physical conditions or under basal levels of TGFβ1 should be explored.

To the best of our knowledge, our study is the first to prove that HMGA1 is dramatically increased in cardiac fibrosis. In cardiac fibrosis, HMGA1 levels are elevated in heart tissues and CFs, related to cardiac fibrosis pathogenesis and cardiac dysfunction. HMGA1 silencing ameliorated aberrant cardiac fibrosis. The regulatory role of HMGA1 in FOXO1 is attributed to its deteriorating effect on cardiac fibrosis. Our studies therefore delineate a novel HMGA1-mediated cardiac fibrosis mechanism and provide new insights into HMGA1-associated therapy in fibrosis disease.

## Data Availability Statement

The original contributions presented in the study are included in the article/Supplementary material, further inquiries can be directed to the corresponding author/s.

## Ethics Statement

The animal study was reviewed and approved by the Institutional Animal Use and Care Committee at the Wuhan University, China.

## Author Contributions

QW and Q-zT conceived and designed the experiments. YY, ZC, QY, and TH performed the experiments. QX and JZ analyzed the data. QW and QX wrote and revised the manuscript. All authors contributed to the article and approved the submitted version.

## Conflict of Interest

The authors declare that the research was conducted in the absence of any commercial or financial relationships that could be construed as a potential conflict of interest.

## Publisher’s Note

All claims expressed in this article are solely those of the authors and do not necessarily represent those of their affiliated organizations, or those of the publisher, the editors and the reviewers. Any product that may be evaluated in this article, or claim that may be made by its manufacturer, is not guaranteed or endorsed by the publisher.
